# Climate Change Does Not Directly Influence Red Squirrel ( *Sciurus vulgaris* ) Distribution in Western Europe

**DOI:** 10.17912/micropub.biology.001589

**Published:** 2025-08-02

**Authors:** Alyson E. V. Buchanan, Phillipa K. Gillingham, Demetra Andreou, Kathy H. Hodder, Melissa A. Toups, Helen Butler, Emilie A. Hardouin

**Affiliations:** 1 Department of Life and Environmental Sciences, Bournemouth University, Poole, England, United Kingdom; 2 Department of Biology, University of Louisiana at Lafayette; 3 Wight Squirrel Project, Isle of Wight, UK

## Abstract

Climate change presents ongoing risks to species like the red squirrel, which, despite its wide range, faces pressures from multiple threats (fragmentation, invasive species, among others). This study assesses the relationship of red squirrel distribution across Western Europe with bioclimatic variables to predict future climate impacts. However, our results suggest that bioclimatic factors have limited predictive power, with no direct impacts identified. Indirect effects, such as the expansion of grey squirrels, may still worsen challenges for red squirrel populations. Addressing these by maintaining habitat quality and connectivity through targeted conservation measures will be crucial for ensuring red squirrel persistence.

**Figure 1. Bioclimatic Influence on Sciurus vulgaris Distribution: Model Performance, Response Curves, and Geographic Variability:**
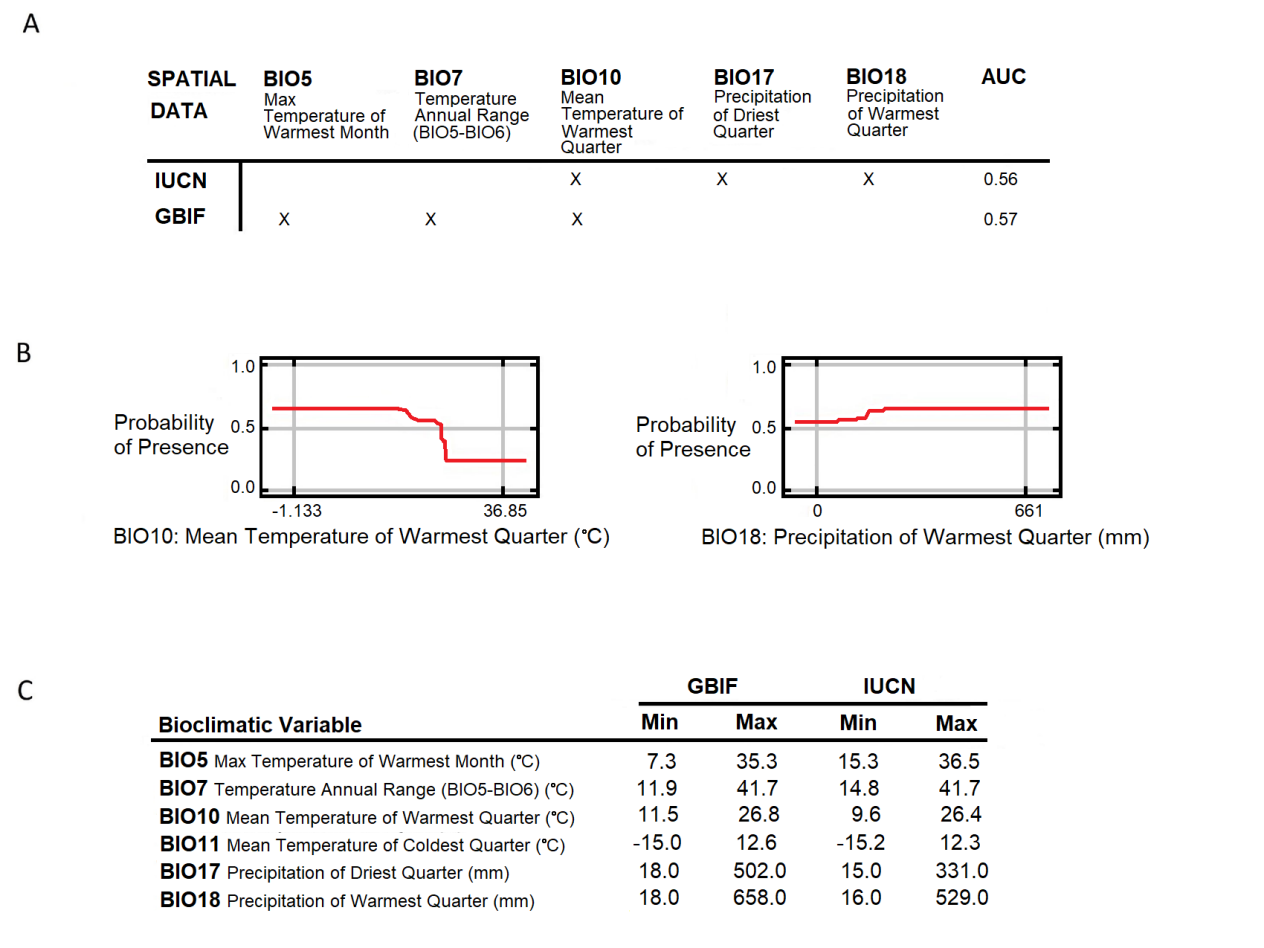
(A) Area Under the Curve (AUC) results for models based on presence data from Western Europe, using current bioclimate variables (1970-2000). An X indicates that bioclimatic variable remained in the best model following variable selection. (B) Response curves for two selected bioclimatic variables, illustrating their relationship with *S. vulgaris* distribution. (C) Minimum and maximum values for key bioclimatic variables across the Western European study area.

## Description

Climate change presents significant risks to biodiversity, especially for species with limited dispersal abilities (Urban 2015; Veron et al. 2019). Species with specialised ecological niches are often more vulnerable to environmental changes, as their capacity to adapt to shifting conditions is limited (Frankham 1997; Leclerc et al. 2020). Generalist species tend to exhibit greater resilience, while specialists frequently experience population declines due to rising temperatures and habitat alterations (Gynther et al. 2016). For instance, Oliveira (2021) showed that rodent species in the Iberian Peninsula respond differently to climate and land-use changes, with generalist species predicted to expand or maintain their ranges, while specialist rodents face range contractions, illustrating the broader pattern observed across taxa. This contrast highlights the varying responses of species to climate change and underscores the importance of understanding how different ecological traits influence vulnerability. Despite being a generalist, the red squirrel ( *Sciurus vulgaris* ) faces significant pressures from environmental change, particularly habitat fragmentation, competition with invasive grey squirrels ( *Sciurus carolinensis* ), and climate change, as evidenced by its declining populations in the UK, where it is classified as endangered on the UK Red List (Gurnell et al. 2004; Levinsky et al. 2007; Mammal Society 2024). The IUCN (2014) lists the red squirrel as Least Concern; however, ongoing climate change could threaten this status, particularly if it enables the expansion of grey squirrels, which are already a threat in regions like the UK and Italy (Bosch and Lurz 2012; Nie et al. 2023).

Red squirrels are widely distributed across Western Europe, occupying diverse habitats ranging from temperate forests to colder boreal regions (Bosch and Lurz 2012; IUCN 2014). They primarily rely on tree seeds from several species, including spruce, pine, hazel, beech, and walnut, though they are flexible and can shift to a variety of other food sources in suboptimal conditions (Gurnell 1987; Bosch and Lurz 2012). Climate-driven changes in forest composition, seed production, and altered phenology may affect food availability and nesting habitat, potentially influencing red squirrel populations (Bosch and Lurz 2012; Flaherty et al. 2012; Reher et al. 2016). Furthermore, shifting temperature and precipitation patterns could alter tree species distributions, affecting the availability of suitable habitats (Razgour et al. 2018).

In addition to habitat changes, invasive species pose a significant threat to red squirrel populations. Grey squirrels, already established in the UK and Northern Italy, are an invasive competitor that competes for food resources and introduces disease (Wauters et al. 2001; Gurnell et al. 2004; Nie et al. 2023; Wauters et al. 2023). Climate change may further facilitate their expansion, increasing competitive pressures on red squirrels, particularly in areas where habitat changes favour grey squirrel survival (Nie et al. 2023). If grey squirrels continue to spread, they could exacerbate the challenges red squirrels face from environmental change.

Moreover, populations in the British Isles, Iberia, and Italy are situated at the geographic margins of the red squirrel’s range. Edge-of-range populations are often more vulnerable to environmental fluctuations due to classic population dynamic factors such as lower densities, reduced genetic diversity, and greater isolation (Lawton 1993; Channell and Lomolino 2000). Recent studies highlight that rear-edge populations may be especially critical for species persistence under climate change, as they can harbour unique genetic diversity and face heightened extinction risks (Hampe and Petit 2005; Sexton et al. 2009).

This study aims to predict the impacts of climate shifts on red squirrel habitat suitability across Western Europe. Although the red squirrel occupies a broad Eurasian range, we constrained our analysis to Western Europe. This decision was originally driven by our focus on the Isle of Wight, where conservation concerns are acute. Initial attempts to model the Isle of Wight directly produced low AUC scores (0.50–0.57), likely due to limited environmental gradients and sparse data. Expanding the model to Western Europe improved robustness while maintaining ecological relevance to the Isle of Wight population. Additionally, attempts to model the full Eurasian range exceeded computational capacity and repeatedly caused system crashes. Western Europe was therefore selected as the most practical and informative extent, large enough to encompass varied climate conditions but focused enough to remain ecologically coherent and technically feasible.

We used Maxent modelling to determine relationships between red squirrel distribution and bioclimatic variables, seeking to identify areas where habitat conditions may become more or less favourable (Guisan and Zimmermann 2000; Razgour et al. 2018). These predictions are crucial for developing effective conservation strategies that address potential consequences of climate change on red squirrel populations (Thuiller et al. 2005; Elith and Leathwick 2009).

Initial Maxent models used bioclimatic data from WorldClim (1970-2000) (Fick and Hijmans 2017) alongside red squirrel presence data. However, the models did not reach an acceptable AUC score (≥0.7), with scores ranging from 0.50 to 0.57 (Fig. 1a), suggesting the selected bioclimatic variables were weak predictors of the species' current distribution (Fielding and Bell 1997). Despite using a refined selection of variables, as indicated by jackknife tests and response curves (Fig. 1b), no single bioclimatic variable emerged as strongly predictive of red squirrel presence. Additionally, the assessed variables showed negligible correlations with their distribution (Fig. 1a), further indicating the models lacked predictive power. Consequently, projecting future distributions under climate change scenarios would be unreliable.

The lack of direct relationships with bioclimatic variables could have several explanations. Red squirrels occupy a wide range of climatic conditions, including both temperate and colder regions (Bosch and Lurz 2012; IUCN 2014), which likely contributes to the weak correlation between climate variables and their current distribution. Wauters et al. (2001) emphasised that habitat quality, particularly food availability and tree species diversity, plays a significant role in determining red squirrel survival. Given the broad climatic range in which red squirrels are found, other ecological factors, such as habitat availability, food resource distribution, and interspecies competition, are likely more influential in shaping their distribution patterns (Delin and Andrén 1999; Gurnell et al. 2004; Romeo et al. 2010; Reher et al. 2016).

Although our study did not find significant direct effects of bioclimatic variables, climate change could still have indirect effects on red squirrel populations. Altered phenology, such as shifts in seed production timing and changes in red squirrel phenology, could disrupt resource availability during critical periods, impacting red squirrel reproduction and survival (Gurnell et al. 2004; Reher et al. 2016). Scots pine, which is more resilient to drought and temperature variability, contrasts with beech, which is more sensitive to warmer, drier conditions (González de Andrés et al. 2017). Climate change may therefore alter the availability of food resources, indirectly influencing red squirrel populations.

Additionally, Nie et al. (2023) modelled grey squirrel range shifts in continental Europe under climate change and found that increased temperatures could extend grey squirrel ranges, potentially leading to greater competition with red squirrels. This highlights the importance of maintaining biosecurity measures across Western Europe to prevent further introductions and mitigate competition, thereby protecting red squirrel populations.

This study underscores the limitations of relying solely on bioclimatic models to predict species distribution, particularly for generalist species, as these models often fail to account for key ecological factors like habitat quality, fragmentation, and interspecies competition, which can play a significant role in shaping their distribution (Pearson and Dawson 2003; Cayuela et al. 2009; Dutra Silva et al. 2019). Conservation strategies should prioritise enhancing habitat quality and connectivity, while preventing the spread of grey squirrels, particularly in regions where they are not yet established. Further research should integrate additional ecological data and test specific interventions, such as reforestation, wildlife corridors, and targeted control measures, to ensure the long-term survival of red squirrels across Western Europe.

Future research could extend the spatial scale of this work to model the entire ranges of both *Sciurus vulgaris* and *Sciurus carolinensis* . Such comparative modelling would help clarify whether climate variables exert differential effects on each species and may reveal how competitive dynamics shift under future climate scenarios. This broader perspective could further inform conservation strategies, especially in areas where both species may come into contact due to climate-driven range shifts.

## Methods

*Bioclimatic Data*

Bioclimatic data were obtained from WorldClim.org, which provides global temperature and precipitation patterns with approximately1km resolution (Hijmans et al. 2005). The datasets included historical climate data from 1970–2000 and future projections for 2080–2100 based on two climate scenarios: SSP1-2.6 (optimistic) and SSP5-8.5 (pessimistic). Three climate models, HadGEM3-GC31-LL, INM-CM5-0, and UKESM1-0-LL, were intended to be used for projections. 19 bioclimatic layers (bio1-19) were extracted from the climate model files using the raster package in RStudio (Hijmans 2023) and then clipped in ArcGIS to focus on Western Europe. Following clipping, the raster layers were converted to ASCII format in ArcGIS to ensure compatibility with Maxent. A correlation matrix was also generated in ArcGIS using the raster attribute tables to identify and remove highly collinear variables prior to modelling. The R script used to extract the bioclimatic raster layers is provided in the extended data (Extended_Data_Rscript.txt).

*Geographic Scope*

Although the red squirrel is distributed throughout Eurasia, our modelling focused on Western Europe. This decision was guided by both methodological and ecological considerations. Our original aim was to model red squirrel habitat suitability specifically on the Isle of Wight, where conservation concerns are particularly acute. However, models built exclusively on the Isle of Wight performed poorly, producing AUC scores below the acceptable threshold (0.50–0.57), likely due to limited climatic variation and sparse presence records at that scale.

To improve the reliability and generalisability of our model, and to determine whether the poor performance on the Isle of Wight was due to genuine ecological limitations or simply the result of a small and homogeneous dataset, we expanded the modelling extent to Western Europe. This scale provided broader bioclimatic variability while retaining ecological relevance to the Isle of Wight population. Initial Western European models produced similar results to those for the Isle of Wight, but with improved performance metrics, validating this broader approach.

Attempts to model the full Eurasian range were not feasible due to system limitations; repeated efforts to run Maxent models on the larger dataset caused system crashes and processing failures. Thus, Western Europe was selected as the optimal compromise between computational feasibility, ecological relevance, and statistical performance.

*Red Squirrel Spatial Records*

Presence data for red squirrels were sourced from the IUCN Red List and the Global Biodiversity Information Facility (GBIF). For the IUCN data, presence points were created in ArcGIS 10.6.1 by converting the Eurasian red squirrel distribution polygon to a raster using *Polygon to Raster* (Conversion) tool with the default resolution. Data from GBIF was filtered to include human observations from 1970–2000, with records within 500 meters of the observed location to match temporal extent and spatial resolution of the bioclimatic data. The presence points were clipped to the study area of Western Europe, resulting in 129,901 records from GBIF and 28,258 from the IUCN polygons. The final presence datasets are provided as extended data (Extended_Data_IUCN.csv and Extended_Data_GBIF.csv).

*Distribution Modelling*

Climate change distribution modelling was conducted using Maxent 3.4.4, a tool that models species distributions based on presence-only data (Phillips et al. 2004). Models were run with 10 replicates, using a 75% training and 25% testing data split, and a random seed was applied to ensure replicability. Model performance was evaluated by averaging the Area Under the Curve (AUC) across replicates. To ensure robustness, environmental variables were selected using a correlation matrix and Maxent’s Jackknife test, which iteratively refined the model to maximise the AUC score, as described by van Gils et al. (2012).

Maxent's background points (10,000 by default) were randomly sampled across the study area of Western Europe without environmental stratification. While this default approach facilitates model training by providing environmental contrast, we acknowledge that it may not fully account for potential sampling bias in presence records. Future studies could explore bias correction techniques or environmentally stratified background sampling to improve model robustness.

Models were also manually re-run multiple times using each of the three climate models (HadGEM3-GC31-LL, INM-CM5-0, and UKESM1-0-LL) and both GBIF and IUCN presence datasets. Several runs used randomly sampled subsets of the IUCN data (e.g., 0.05%, 0.5%, 10%, 20%) to evaluate sensitivity to sampling intensity. Across all variations, models consistently produced low AUC values and weak response patterns, indicating that bioclimatic variables alone do not adequately explain the species distribution. These findings support the appropriateness of using standard evaluation methods such as AUC and response curves in this study.

## Data Availability

Description: R script extended data. Resource Type: Text. DOI: https://doi.org/10.22002/fabt6-mnv08 Description: IUCN sourced red squirrel presence data. Resource Type: Dataset. DOI: https://doi.org/10.22002/tc3n2-b0d23 Description: GBIF sourced red squirrel presence data. Resource Type: Dataset. DOI: https://doi.org/10.22002/qvxvh-r4w60
